# Receptor depletion and recovery in small-intestinal neuroendocrine tumors and normal tissues after administration of a single intravenous dose of octreotide measured by ^68^Ga-DOTATOC PET/CT

**DOI:** 10.1186/s13550-021-00860-0

**Published:** 2021-11-25

**Authors:** Ulrika Jahn, Ezgi Ilan, Irina Velikyan, Katarzyna Fröss-Baron, Mark Lubberink, Anders Sundin

**Affiliations:** 1 Radiology and Molecular Imaging, Department of Surgical Sciences, Uppsala University, Uppsala, Sweden; 2 Uppsala University Hospital, 75185 Uppsala, Sweden; 3 Medical Physics, Uppsala University Hospital, Uppsala, Sweden; 4 Medical Imaging Centre, Uppsala University Hospital, Uppsala, Sweden

**Keywords:** ^68^Ga-DOTATOC, PET/CT, Peptide, Small-intestinal NET, Receptor internalization, SUV, K_i_

## Abstract

**Background:**

Low-grade neuroendocrine tumors (NETs) are characterized by an abundance of somatostatin receptors (SSTR) that can be targeted with somatostatin analogs (SSA). When activated with a single dose of SSA, the receptor-ligand complex is internalized, and the receptor is by default recycled within 24 h. Ongoing medication with long-acting SSAs at ^68^Ga-DOTA-SSA-PET has been shown to increase the tumor-to-normal organ contrast. This study was performed to investigate the time-dependent extended effect (7 h) of a single intravenous dose of 400 µg short-acting octreotide on the tumor versus normal tissue uptake of ^68^Ga-DOTATOC.

**Methods:**

Patients with small-intestinal NETs received a single intravenous dose of 400 µg octreotide and underwent dynamic abdominal ^68^Ga-DOTATOC-PET/CT at three sessions (0, 3 and 6 h) plus static whole-body (WB) PET/CT (1, 4 and 7 h), starting each PET/CT session by administering 167 ± 21 MBq, 23.5 ± 4.2 µg (mean ± SD, *n* = 12) of ^68^Ga-DOTATOC. A previously acquired clinical whole-body ^68^Ga-DOTATOC scan was used as baseline. SUV and net uptake rate K_i_ were calculated in tumors, and SUV in healthy organs.

**Results:**

Tumor SUV decreased significantly from baseline to 1 h post-injection but subsequently increased over time and became similar to baseline at 4 h and 7 h. The tumor net uptake rate, K_i_, similarly increased significantly over time and showed a linear correlation both with SUV and tumor-to-blood ratio. By contrast, the uptake in liver, spleen and pancreas remained significantly below baseline levels also at 7 h and the receptor turn-over in tumors thus exceeded that in the normal tissue, with restitution of tumor ^68^Ga-DOTATOC uptake mainly completed at 7 h. These results however differed depending on tumor size, with significant increases in K_i_ and SUV between the 1st and 2nd PET, in large tumors (≥ 4 mL) but not in small (> 1 to  < 4 mL) tumors.

**Conclusion:**

SSTR recycling is faster in small-intestinal NETs than in liver, spleen and pancreas. This opens the possibility to protect normal tissues during PRRT by administering a single dose of cold peptide hours before peptide receptor radionuclide therapy (PRRT), and most likely additionally improve the availability and uptake of the therapeutic preparation in the tumors.

**Supplementary Information:**

The online version contains supplementary material available at 10.1186/s13550-021-00860-0.

## Background

Neuroendocrine neoplasms (NENs) constitute a heterogenous group of tumors originating in the diffuse endocrine system of cells and disseminated throughout the body [1]. The well-differentiated neuroendocrine tumors (NETs) are divided according to their proliferation into grade 1 (Ki-67 index ≤ 2%) grade 2 (Ki-67 index 3–20%), grade 3 (Ki-67 index > 20%), and the grade 3 tumors are further divided into low-differentiated grade 3 neuroendocrine cancers (NECs). NENs bare traits of both neuroendocrine characteristics and that of the specific tissue where they originate [2–4]. The SI-NETs mainly express somatostatin receptor (SSTR) subtype 2 to about 80% [5–8], and to a lesser extent subtypes 3 and 5 of the G-protein-coupled (GPCR) trans-membrane type, that are encoded on separate genes (SSTR_2_/17, SSTR_3_/22, and SSTR_5_/16) [9–11]. Upon activation, the receptor/ligand complex is rapidly depleted from the outer membrane through internalization and to a large extent dissociated, with a half-time of approximately 10 min [12]. The SSTR_2_ receptor is then by default recycled through various intracellular pathways from the early to the late endosomes [13]. After budding off from the late endosomes, the SSTR_2_ either enters the trans-Golgi system and the perinuclear region, or returns to the cell membrane [13, 14]. After a single activation of the receptors, the majority internalize and recycle to the cell surface within 16 to 24 h, as the low mRNA activity indicate a negligible de novo receptor synthesis [15, 16]. Only for SSTR_5_, there is an internal pool of receptors that is rapidly activated and transported to the cell membrane upon stimulation [17]. In the early days of ^111^In-pentetreotide-scintigraphy, there was a concern that medication with octreotide would hamper the imaging results due to the competitive binding of the radio-labeled and unlabeled analogs to the receptors. However, Dörr et.al. reported that SSA medication did not change the diagnostic accuracy of a somatostatin receptor scintigraphy, but quite the opposite; by internalizing the receptors to a relatively greater extent in the normal tissues than in the tumors, the tumor-to-normal tissue contrast, and thereby the tumor detection, was improved [18]. Based on their finding, they further suggested, in the context of peptide receptor radionuclide therapy (PRRT), that administration of cold peptide might even improve the tumor uptake of the therapeutic preparation [18]. Continuation of SSA medication has also proven favorable for NET imaging with ^68^Ga-DOTA-SSA-PET/CT [19–22]. In a previous study from our group, intravenous injection of 50 µg SSA decreased the normal tissue uptake and increased the tumor uptake of ^68^Ga-DOTATOC, increasing the tumor-to-normal tissue contrast, whereas 500 µg of intravenous SSA further decreased the normal tissue uptake, and also that in the tumors, except in a large pancreatic NET with very high SSTR expression [23].

The present study aimed to analyze the time-dependent extended effect of receptor depletion and recirculation during 7 h, in SI-NET metastases and in normal tissues, following a single intravenous dose of short-acting SSA (400 µg octreotide), by means of three serial ^68^Ga-DOTATOC-PET/CT examinations in the same patients.

## Methods

### Patients

**Fig. 1:**
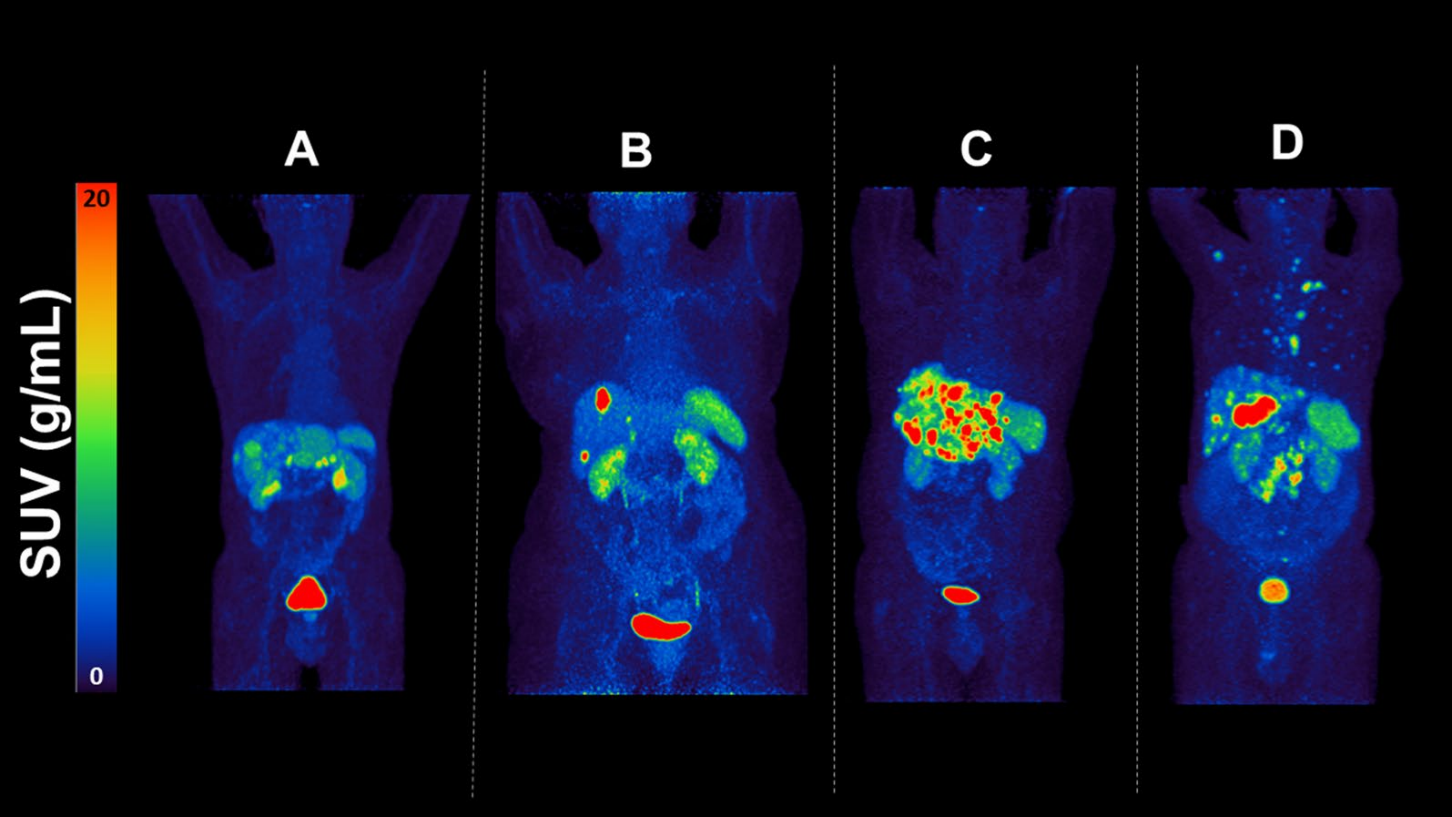
Maximum intensity projections of patient 1–4 (**A**–**D**) at baseline whole-body ^68^Ga-DOTATOC showing SUV values, where red color represents SUV of 20 and blue SUV of 0

Four patients with disseminated small-intestinal NETs, who were all progressing on long-acting SSA (Table 1) and scheduled for PRRT at University Hospital, Uppsala, Sweden, were included. The study was approved by the Regional Ethics Committee (No. 2014/39), and each patient provided a written informed consent. All procedures were performed in accordance with the 1964 Helsinki declaration and its later amendments and comparable ethical standards. ^68^Ga-DOTATOC-PET/CT performed to assess the patients’ eligibility for PRRT was used as the baseline examination of reference. All patients harbored liver metastases (patients 1, 3 and 4 approximately 20–25%, and patient 2 < 5% of the liver), bone metastases and retroperitoneal lymph node metastases were also present in patient 4 (Table 1). Thus, the tumor burden was low in patient 2, intermediate in patients 1, 3 and 4, and ranked in the order highest to lowest 3, 4, 1 and 2. Maximum intensity projections at baseline whole-body ^68^Ga-DOTATOC PET are presented in Fig. 1.

**Table 1:** Basic patient characteristics, SSA medication, dosing interval and interval between SSA administration and ^68^Ga-DOTATOC PET/CT examinations Pat: patient, M: male, F: female, w: weeks, d: days, mets: metastases

Pat	Age	Sex	SSA medication	Time (d) between SSA and ^68^Ga-DOTATOC PET/CT Baseline	Time (d) between SSA and ^68^Ga-DOTATOC-PET/CT Study	Tumor burden*
1	74	M	Sandostatin LAR 30 mg/3 w	17	17	Liver mets 20–25%
2	73	F	Santostatin Autogel 120 mg /2 w	14	13	Liver mets < 5% lgll. mets, bone mets,
3	61	M	Santostatin Autogel /120 mg 2 w	7	6	Liver mets 20–25%
4	68	M	Santostatin Autogel 120 mg /4 w	27	25	Liver mets 20–25%

*Tumor burden was estimated on the baseline ^68^Ga-DOTATOC-PET/CT

### PET examinations

The day before start of PRRT was set as the study day for the three ^68^Ga-DOTATOC PET/CT serial examinations, while all the patients still were on medication with long-acting SSA. Because of logistical reasons, the interval between the baseline ^68^Ga-DOTATOC-PET/CT examination and the study examinations varied (3.5, 2.5, 1.5 and 1.0 months, respectively, for patients 1–4). The time interval between long-acting SSA administration and the baseline ^68^Ga-DOTATOC-PET/CT examination, and that between long-acting SSA administration and the study examinations were almost similar (Table 1). On the study day, 400 µg of octreotide in 0.5 L 0.9% of physiological saline was administered as an intravenous infusion during 10–15 min immediately after which the first injection of ^68^Ga-DOTATOC was administered through a second intravenous catheter during 10 s using an automatic injector. All examinations were performed on the same digital time-of-flight PET/CT scanner (Discovery MI, GE Healthcare, Milwaukee Wisconsin, USA). The dynamic ^68^Ga-DOTATOC-PET/CT examinations were performed in three sessions at time 0 (1st dynamic scan), 3 h (2nd dynamic scan) and 6 h (3rd dynamic scan), starting simultaneously with the intravenous injection of 2 MBq/kg ^68^Ga-DOTATOC, mean ± SD 167 ± 21 MBq and 23.5 ± 4.2 µg (Patient 1: 148–196 MBq, 18.5–23.6 µg, patient 2: 161–172 MBq, 18.4–19.7 µg, patient 3; 196–205 MBq, 25.3–29.6 µg and patient 4: 149–152 MBq, 27.7–27.9 µg). The dynamic scans were acquired in list mode for 45 min and reconstructed in 22 time frames (6 × 10 s, 3 × 20 s, 3 × 60 s, 5 × 180 s, 5 × 300 s). Following a short break, a whole-body PET/CT was performed from the proximal thighs to the base of the skull, 3 min per bed-position, at time 1 h (WB 1), 4 h (WB 2) and 7 h (WB 3).

Immediately before session 2 and 3, a 5 min static acquisition of the upper abdomen (corresponding to the field-of-view for that of the dynamic PET examinations) was performed to account for remaining activity from previous sessions. A low-dose CT examination was performed at each session for attenuation correction of the PET images. All appropriate corrections were applied to the PET data, and the PET images were reconstructed using time-of-flight OSEM (Ordered Subset Expectation Maximization) with 3 iterations and 16 subsets, including resolution recovery, and a 5-mm gaussian post-processing filter and a matrix size of 256 × 256 with a reconstructed FOV of 50 cm.

### Radiochemistry

^68^Ga was obtained from a pharmaceutical grade ^68^Ge/^68^Ga generator (GalliaPharm®, Eckert & Ziegler, loaded ^68^Ge of 1850 MBq) eluted with 0.1 M hydrochloric acid solution. ^68^Ga-DOTATOC was produced using an automated synthesis platform (Modular PharmLab, Eckert & Ziegler, Germany) equipped with a disposable cassette system (C4-Ga68-PP). The product was eluted with 1 mL of 50% EtOH and formulated in sterile sodium chloride (0.9%). The subsequent sterile filtration of the product was conducted in-line. Radiopharmaceutical release specifications such as radiochemical purity, chemical purity, quantity, solution color and clarity were controlled. The quality control in terms of purity and peptide content was conducted using high-performance liquid chromatography (HPLC) with UV- and radio-detectors connected in series. The amount of the peptide determined using HPLC calibration plot as a part of quality control and the respective radioactivity amount were used to calculate the specific activity (SRA, MBq/µg) value that was thereafter used to calculate the amount of the peptide injected to a patient based on the amount of the injected radioactivity (Eq. 1). The radioactivity values were decay corrected to the radiopharmaceutical administration time point.

1 $${\text{Injected}}\;{\text{mass}} ({\mu g}) = \frac{{{\text{Injected}}\;{\text{radioactivity}}\;({\text{MBq}})}}{{{\text{SRA}}\left( {\frac{{{\text{MBq}}}}{{{\mu g}}}} \right)}}$$

### PET measurements

The 5 min static acquisition image acquired before WB 2 and WB 3 was used to correct for remaining activity from previous sessions. In all four whole-body examinations (WB 0, 1, 2 and 3), the mean standard uptake value (SUV) was measured in tumors with diameter lager than 1 cm in diameter and by choosing those with the highest tracer uptake (determined visually) by applying a 50% iso-contour volume of interest (VOI) (Hybrid Viewer PDR version 5.1.1. Hermes Medical Solutions, Stockholm). For normal organs, liver, right and left kidney, spleen, pancreas and bone marrow, mean SUV was determined by placing a spherical VOI (1.5 cm in diameter) in a representative healthy part of the organ. Whole blood uptake (mean SUV) was measured by placing a spherical VOI (1 cm in diameter) in the left ventricle of the heart.

Kinetic analysis was performed for the dynamic images acquired at session 1, 2 and 3. Iso-contour VOIs (50% of the maximum activity) were drawn on the 20- to 45-min (frames 18–22) summation image of the dynamic PET image (Hybrid Viewer PDR) and the VOIs were then transferred to all time frames in the dynamic examination generating a tumor time-activity concentration curve. The total radioactivity concentration in the arterial plasma was used as the input function. A spherical VOI (1 cm in diameter) was placed over the descending thoracic aorta in the 0- to 180-s (frames 1–10) summation image of the dynamic PET image, in which the first passage of the bolus was best visualized. This was then projected onto all time frames generating an arterial time-activity concentration curve. The image derived input functions were calculated as described previously [24] by multiplying the arterial time–activity concentration curve with a fixed plasma-to-whole-blood ratio of 1.6. The net influx rate, K_i_, was determined using the Patlak method [25] as previously detailed [26].

Mean tumor SUV and tumor-to-blood ratio (TBR) were also computed for the last frame of the dynamic scan (i.e., 40–45 min post. injection) on all three scans. The tumor uptake was determined by using a 50% iso-contour VOI and the uptake in blood using a 1 cm VOI placed in the descending aorta.

Tumor measurements was also performed using only the two largest tumors of each patient. The tumors were also separated into small and large tumors based on the 50% iso-contour VOI on PET, with an arbitrary limit at 4 mL where large tumors were classed ≥ 4 mL and small tumors were larger than 1 (> 1) mL but smaller than 4 (< 4) mL.

### Statistical methods

Mean tumor and organ SUV were compared between WB 0, 1, 2 and 3 using a Wilcoxon matched-pairs signed rank test with a significance level set to *P* less than 0.05 (Prism, version 6.07; GraphPad Software, Inc.). Tumor and organ SUV in WB 1, 2 and 3 were also normalized against WB 0, and a Wilcoxon matched-pairs signed rank test (significance level set to *P* less than 0.05) was applied. K_i_ was determined for the dynamic acquisitions, and the relation between the three dynamic acquisitions was evaluated using a Wilcoxon matched-pairs signed rank test (significance level set to *P* less 0.05). The relation between K_i_ and TBR was evaluated using linear regression and Pearson correlation and compared with the relation between K_i_ and SUV.

Whole blood uptake (mean SUV) was also compared between WB 0, 1, 2 and 3 using a Wilcoxon matched-pairs signed rank test (significance level set to *P* less than 0.05).

## Results

### PET results: tumors

**Fig. 2:**
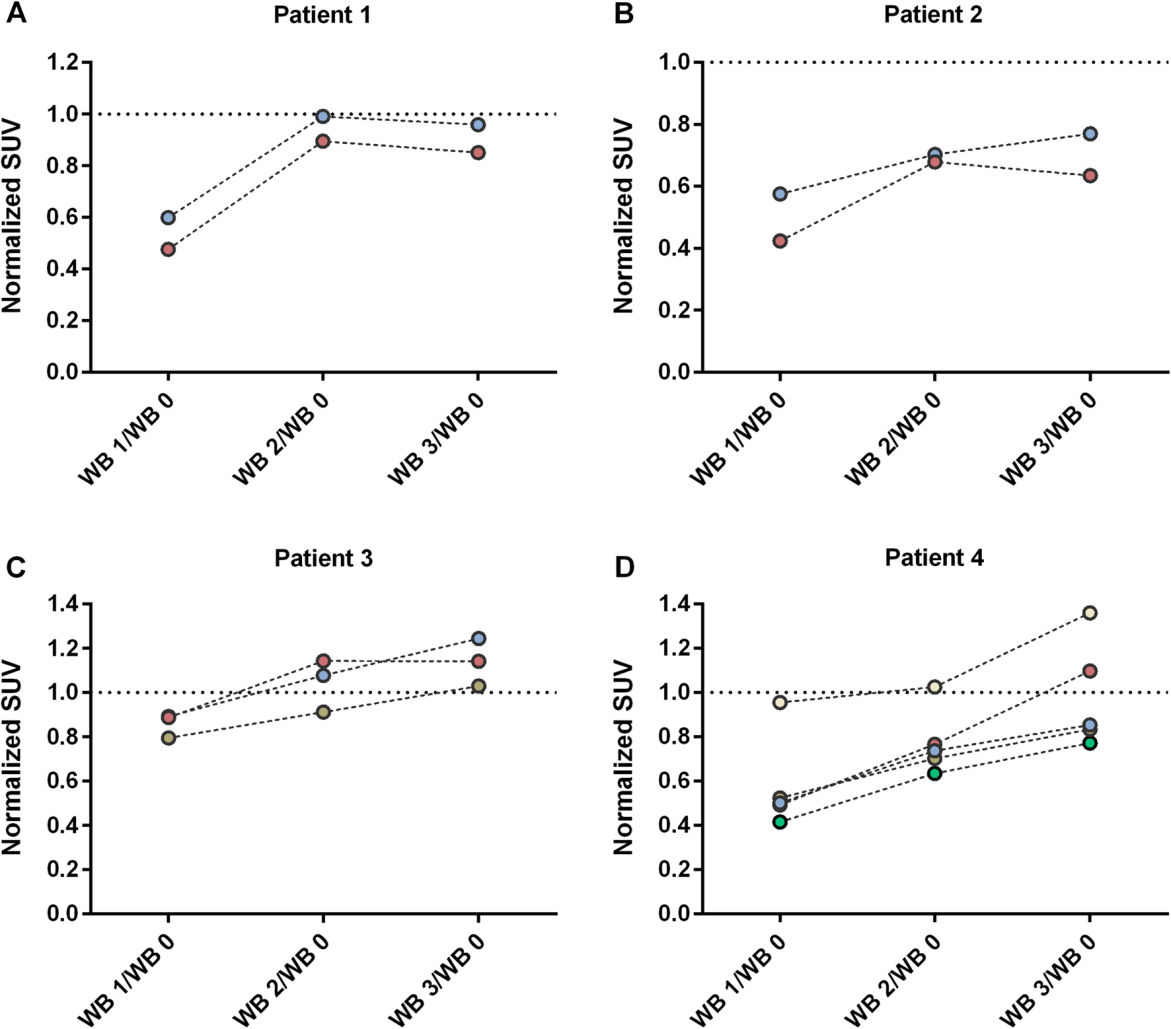
Normalized tumor SUV, where SUV at WB 1 (0 h), WB 2 (4 h) and WB 3 (7 h) are normalized against WB 0 (SUV ratio) for patient 1 (**A**), patient 2 (**B**), patient 3 (**C**) and patient 4 (**D**). Each color represents one tumor and the dotted line represents a normalized SUV of 1

**Fig. 3:**
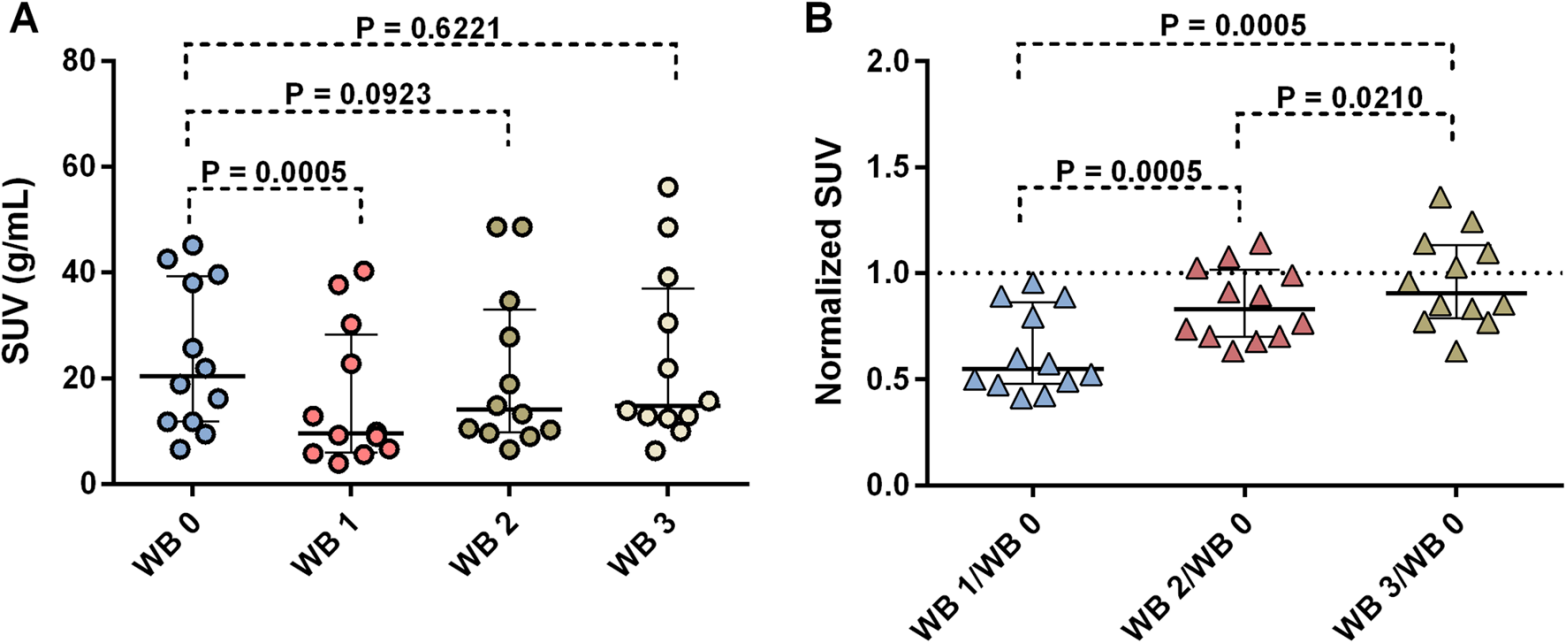
Scatter dot plots for (**A**) mean tumor SUV at baseline (WB 0), WB 1 (0 h), WB 2 (4 h) and WB 3 (7 h) and (**B**) normalized SUV, whereby the tumor SUV at WB 1, 2 and 3 is normalized against tumor SUV at WB 0 (SUV ratio). The solid line represents median and interquartile range and the dotted line (**B**) normalized SUV value of 1. Significant decrease (*P* < 0.05) was found in tumor SUV between WB 0 and WB 1, however, not between WB 0 and WB2 and WB 0 and WB 3 (*P* > 0.05). Significant subsequent increase was found in normalized SUV between all three WB examinations (WB 1, 2 and 3) (**B**). Thus, the normalized SUV returned to baseline levels at 4 and 7 h

SUV and K_i_ were measured for twelve tumors in four patients—Patient 1 and 2 had two tumors each, patient 3 had three tumors and patient 4 had five tumors. The mean tumor SUV in WB 1 (0 h), WB 2 (4 h) and WB 3 (7 h) was normalized against SUV in the baseline examination (WB 0). As illustrated in Fig. 2, the tumor uptake decreased between WB 0 and WB 1 (normalized SUV < 1). At WB 2 and WB 3, the tumors demonstrated a significant recovery of SSTR activity back to the baseline values, or even above, at the last time point after the 400 µg Octreotide injection (Fig. 2). In the analysis of all tumors, between WB 0 and WB 1 (P = 0.0005) a significant decrease was found in tumor SUV, however, not between WB 0 and WB 2 (P = 0.0923) and WB 3 (P = 0.6221), respectively (Fig. 3A). When normalizing tumor SUV in WB 1, 2 and 3 against SUV in WB 0, a significant increase was found both between WB 1/WB 0 and WB 2/WB 0 (P = 0.0005), between WB 1/WB 0 and WB3/WB 0 (P = 0.0005) and between WB 2/WB 0 and WB 3/WB 0 (P = 0.0210), (Fig. 3B). Tumor SUV for each patient during baseline (WB 0), WB 1(0 h), WB 2(4 h) and WB 3(7 h) is presented in Additional file 1: Figure S1. Separate analyses of the largest two tumors of each patient followed the same pattern (Additional file 2: Figure S2). Assessing small (> 1 to  < 4 mL) versus large (≥ 4 mL) tumors, however, demonstrated that tumor SUV in small tumors showed no significant change (*P* > 0.05) between WB 1 and WB 2 and 3, respectively, whereas in large tumors, a significant decrease was found between WB 1 and WB 2 (*P* < 0.05) (Additional file 3: Figure S3).

Regarding normalized SUV, the large tumors followed the same receptor depletion and recovery pattern as all tumors, with significant raise between WB 1 (0 h) and WB 2 (4 h) and between WB 1 (0 h) and WB 3 (7 h) while the small tumors showed no significant change (Additional file 4: Figure S4).

**Fig. 4:**
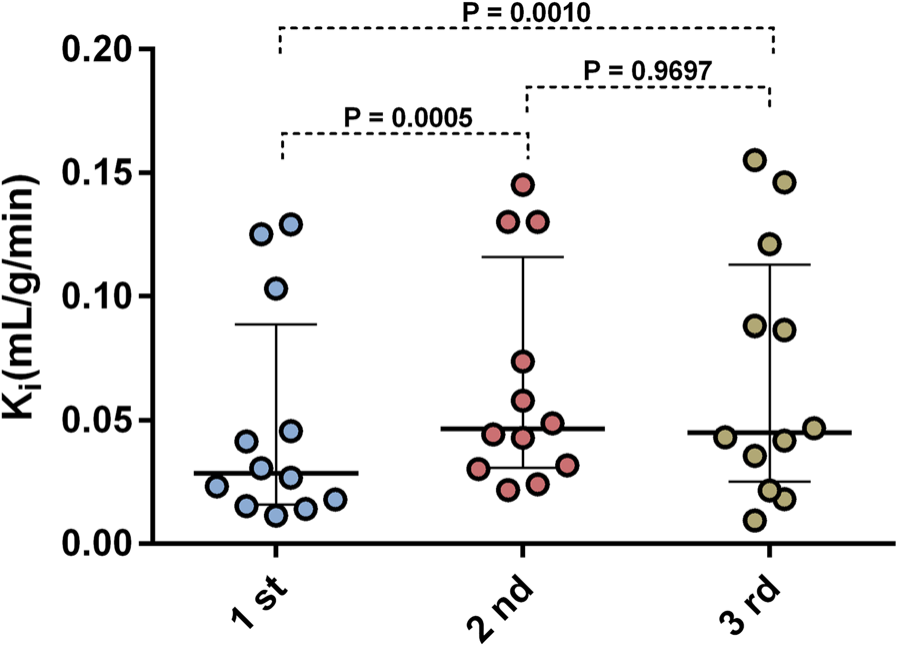
Scatter dot plots of tumor K_i_ at the first (0 h), second (4 h) and third (7 h) dynamic scan. The solid line represents median and interquartile range. Significant subsequent increases (*P* < 0.05) were found in tumor K_i_ between the first and second and first and third dynamic scan, however, not between second and third scan (*P* > 0.05)

Net uptake rate, K_i_, was determined from the dynamic images using the Patlak method [25]. A significant increase in tumor K_i_ between first and second (*P* = 0.0005) and first and third (*P* = 0.0010) dynamic scan was shown (Fig. 4). A statistical test limited to the two largest tumors in each patient showed similar results (Additional file 5: Figure S5). Separate analyses of small (> 1 to  < 4 mL) and large (≥ 4 mL) tumors, respectively, showed that tumor K_i_ in large tumors followed the same receptor depletion and recovery pattern as all tumors, whereas the small tumors did not (Additional file 6: Figure S6).

### PET results: normal organs

**Fig. 5:**
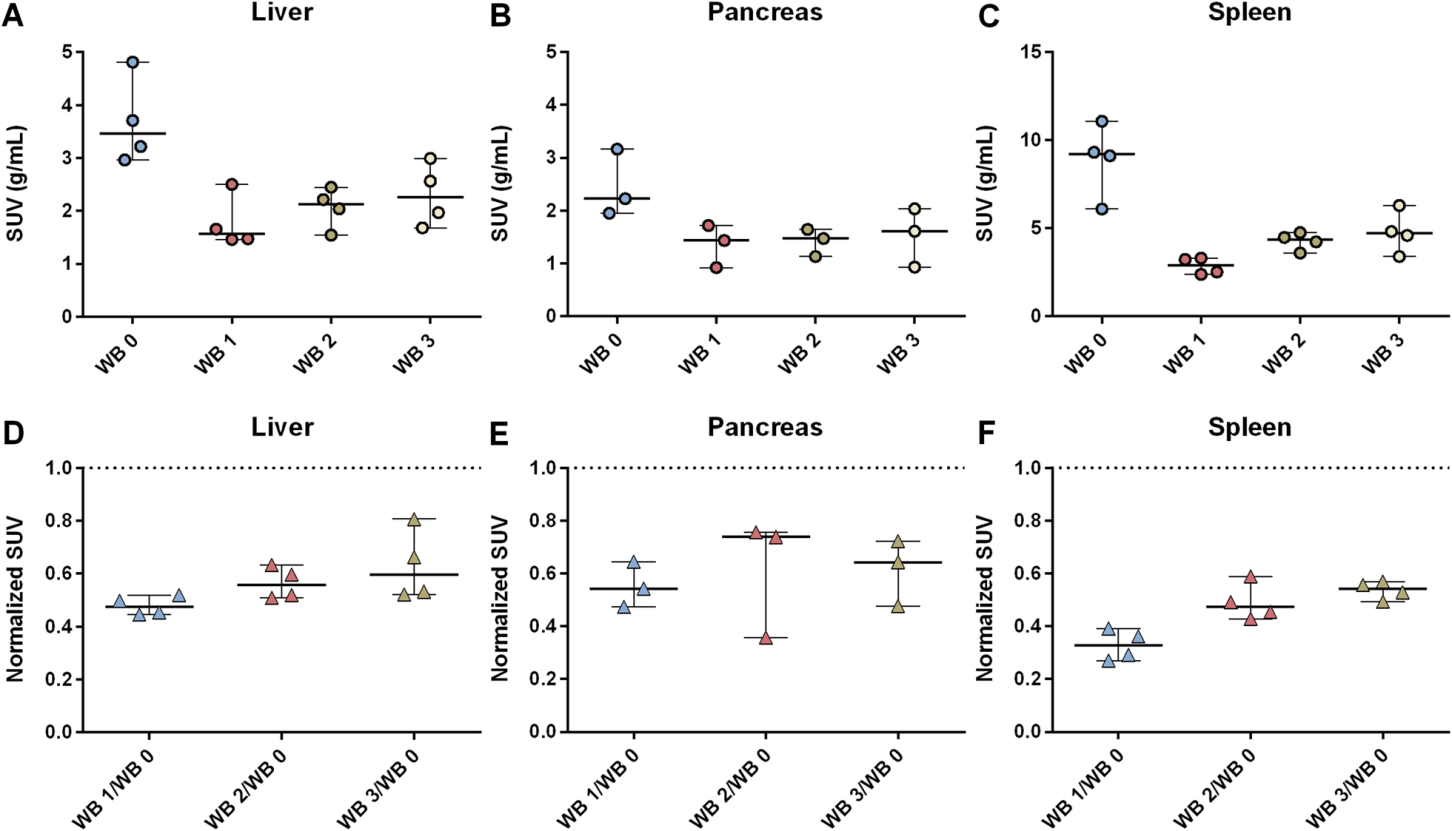
Scatter dot plots of SUV at baseline (WB 0), WB 1 (0 h), WB 2 (4 h) and WB 3 (7 h) in (**A**), liver (**B**) pancreas and (**C**) spleen. Normalized SUV, where SUV in WB 1, 2 and 3 is normalized against SUV at WB 0 (SUV ratio) in (**D**) liver, (**E**) pancreas and (**F**) spleen. The solid line represents median and range and the dotted line the normalized SUV value of 1. Following the initial SUV drop from baseline (WB 0) to WB1 (0 h), a slow recovery was found during WB 2 (4 h) and WB 3 (7 h), but not back to baseline levels

Mean SUV was measured in all parenchymal abdominal organs and in the bone marrow except for the pancreas in patient no.1, which was almost totally replaced by fat and harbored metastases. In the baseline examination (WB 0), the highest SUV was found in the spleen, with a median (range) SUV of 9.2 (6.1–11.1), (Fig. 5C). After the 400 µg octreotide injection, SUV in liver, pancreas and spleen decreased as compared to baseline (Fig. 5A-C), but not in the kidneys or bone marrow (Additional file 8: Figure S8). SUV in liver, pancreas and spleen slowly recovered during WB 2 (4 h) and WB 3 (7 h) but not back to the baseline levels within the 7 h time frame of the study (Wilcoxon matched-pairs signed rank test, *P* > 0.05). The SUV development in normal organs for each patient is presented in Additional file (Additional file 7: Figure S7). The kidneys and the bone marrow lacked this SUV drop, and recovery pattern was similar between examinations. The normalized SUV in liver, pancreas and spleen remained below one (Fig. 5D-F), whereas, for kidney and spleen, some patients showed a normalized SUV ≥ 1, with higher SUV in WB 1, 2 and 3, exceeding that at baseline (WB 0) (Additional file 8: Figure S8).

### Net uptake rate K_i_ versus SUV and tumor-to-blood ratio (TBR)

**Fig. 6:**
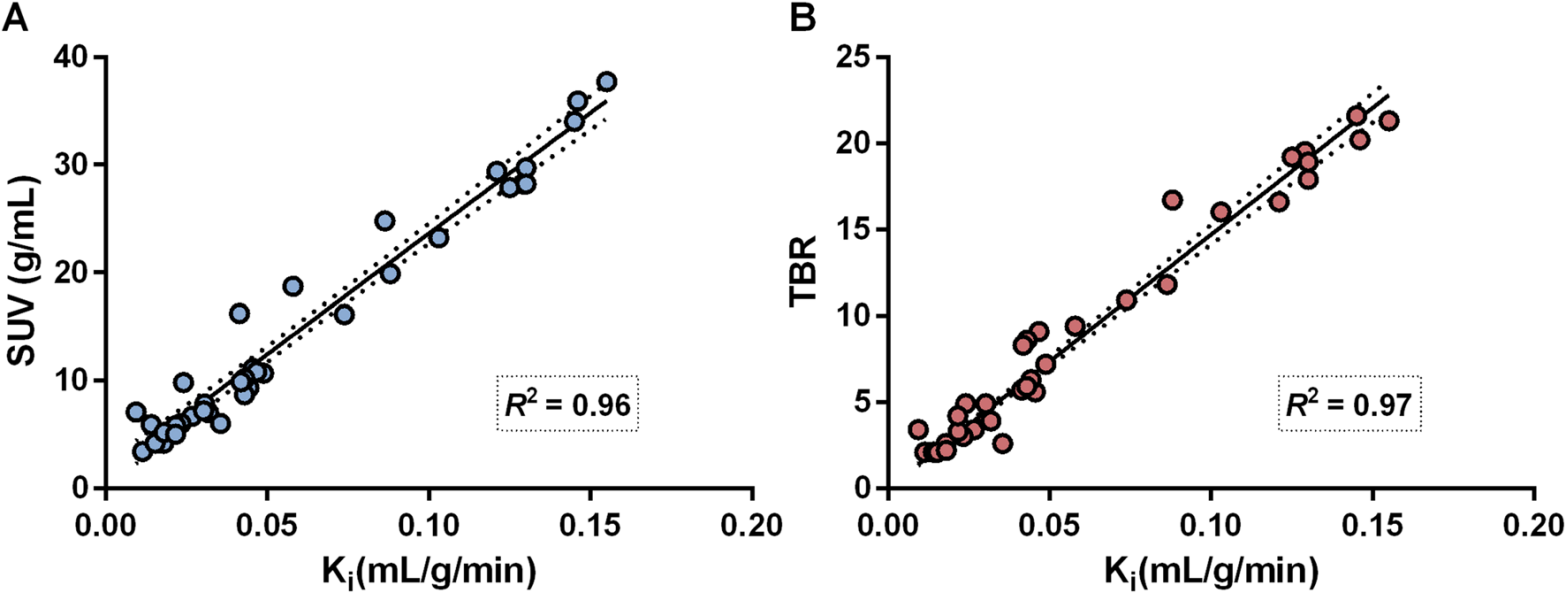
Correlation between (**A**) tumor K_i_ and tumor SUV and between (**B**) tumor *K*_*i*_ and tumor-to-blood ratio (TBR) during all three dynamic scans. Solid line represents linear regression fits and dashed lines are 95% confidence band of these fits. *R*^2^ represents the square of Pearson correlation

A linear correlation was found between K_i_ and SUV and K_i_ and TBR, with a square of Pearson correlation (*R*^2^) of 0.96 and 0.97, respectively (Fig. 6). The figure illustrates the correlation between K_i_ vs SUV and K_i_ vs TBR during all the three sessions. The correlations between K_i_ and SUV and K_i_ and TBR were also evaluated including the two largest tumors per patient (Additional file 9: Figure S9). The relation between K_i_ and SUV, or K_i_ and TBR was similar in all three dynamic examinations. Separate analyses of small (> 1 to  < 4 mL) and large (≥ 4 mL) tumors, respectively, did not affect the correlation between K_i_ and SUV (Additional file 10: Figure S10) or K_i_ and TBR (Additional file 11: Figure S11). The net uptake rate, K_i_, for each tumor per patient is presented in the Additional file (Additional file 12: Figure S12).

### Blood measurements

Whole blood uptake (mean SUV blood) was compared between baseline (WB 0), WB 1 (0 h), WB 2 (4 h) and WB 3 (7 h) that was found similar between examinations (Wilcoxon matched-pairs signed rank test (*P* > 0.05)).

### CT measurements: tumors

The tumor sizes at WB1 (0 h) (the day before start of PRRT), those at last follow-up, and their percentage change are shown in Table 2.

**Table 2:** Size and change of reported tumors according to contrast-enhanced CT

Tumor	Baseline diameter (mm)	Last follow-up diameter (mm)	Change (%)	Time from WB1 to last follow-up (months)
1:1	76*^	75	− 2	11
1:2	20^	19	− 5	11
2:1	30*^	19	− 37	5
2:2	17*^	15	− 12	5
3:1	22	21	− 5	4
3:2	20^	20	0	4
3:3	23^	23	0	4
4:1	57*^	54	− 6	4
4:2	(Rib metastasis)	(Rib metastasis)		
4:3	23^	21	− 9	4
4:4	19	17	− 11	4
4:5	14	15	+ 7	4

*Tumors with patchy areas of necroses

^Tumors selected for the separate analyses of the two largest tumors per patient (Please see Additional file 3: Figure S3, Additional file 4: Figure S4 and Additional file 6: Figure S6)

## Discussion

We assessed the kinetics of SSTRs reappearance in metastatic SI-NETs compared to that in the normal parenchymal abdominal organs (liver, spleen, pancreas and kidneys) and in bone marrow (vertebral body), after a single i.v. 400 µg dose of short-acting octreotide by means of serial ^68^Ga-DOTATOC-PET/CT.

We assessed all tumors with a functional volume larger than 1 mL in order to achieve reasonable precision in the PET measurements, since quantification becomes very inaccurate for lesions below this size due to partial volume effects, and test–retest reproducibility is thus impaired for smaller lesions.

As compared to the baseline examination, the vastly predominant tumor SSTR response pattern was a significant drop in SUV after the 400 µg octreotide injection on the first ^68^Ga-DOTATOC-PET/CT (WB 1), as well as in liver, spleen and pancreas. Over time, in the two subsequent ^68^Ga-DOTATOC-PET/CT examinations (WB 2 (4 h) and WB 3 (7 h)), SUV was restored to the baseline levels, in almost all tumors (Figs. 2 and 3), whereas only a partial recovery was shown in liver, spleen and pancreas, at 7 h (the last point of measurement), (Fig. 5).

The separate analyses limited to the two largest tumor per patient showed similar results (Additional file 2: Figure S2, Additional file 5: Figure S5, Additional file 9: Figure S9). When additionally assessing small (> 1- < 4 mL) and large (≥ 4 mL) tumors, separately, it showed that tumor SUV and normalized SUV in large tumors followed the same receptor depletion and recovery pattern as all tumors, whereas the small tumors similarly showed a decrease following octreotide injection, but not reaching statistical significance (*P* = 0.06) (Additional file 3: Figure S3 and Additional file 4: Figure S4). This may be related to the problems with PET measurements in small objects, as discussed above, few tumors in each group (5 versus 7) in the statistical testing, but also to tumor biology.

Our finding of SSTR depletion and recovery was in agreement with Froidevaux et al., who in an in vivo tumor mouse model demonstrated that 80% of SSTR disappeared from the tumor cell surface within 0.5 h, although a total receptor recovery in their model required 24 h [16]. Also, our results were consistent with previous findings that ongoing medication with long-acting SSA decreases the normal tissue uptakes and does not apparently affect the tumor uptake of ^68^Ga-DOTATATE and thus improves tumor detection [20–22]. It has earlier been demonstrated in a mouse tumor model that the endocytosed receptors are predominantly recycled, and that the de novo synthesis is low for SSTR_1_- SSTR_4_ [16], whereas SSTR_5_ is the only receptor which is stored intracellularly and can rapidly be recruited [17]. Further, ^68^Ga-DOTATOC is known to bind both to SSTR_2_ and SSTR_5_ [27]. It may be hypothesized that the present finding of a rapid rebound effect of the tumor SSTR expression could partly be explained by a larger intracellular pool of SSTR_5_ in tumors as compared to normal tissues, which upon stimulation are recruited in order to restore the membrane pool of SSTR. Moreover, tumors and normal tissues may differ in the de novo syntheses and the degradation activity of the ligand-receptor complex [28, 29]. Yet, internalization and cellular recycling of tumor SSTR might proceed otherwise in the clinical situation, compared to what is known from in vitro systems [13, 14, 30].

The dynamic examinations provided a means to determine the tumor net influx rate, K_i_, which was found to change in parallel with the tumor SUV over time. An interesting finding was the strict correlation between K_i_ and SUV, even for tumors with a high uptake. This was opposed to our previous results in patients with gastro-entero-pancreatic NETs, for which this relationship became nonlinear for pancreatic NETs with SUV > 25, corresponding to K_i_ 0.2, showing that SUV for these tumors was not an accurate measurement of SSTR expression [24, 31]. However, in our present study in exclusively SI-NET patients, the tumor K_i_, in spite of high SUV values > 25, was below 0.2, for which the K_i_ versus SUV relationship was still linear. There are two possible explanations for this difference. Firstly, in Velikyan et al., the high tumor burden resulted in extremely low tracer availability in blood already shortly after injection, putting a limit to tumor SUV values, whereas the tumor burden was much lower in the SI-NET patients included in the present study. Secondly, our administration of 400 µg octreotide, as compared to 20 µg in Velikyan et al., results in higher blood tracer concentration due to depletion of binding sites in healthy organs and tumors, allowing SUV to keep up with K_i,_ even at high K_i_ values (Fig. 6). The kidneys and bone marrow failed to respond to the i.v. octreotide injection with a decrease in the ^68^Ga-DOTATOC uptake, showing similar SUV in all three subsequent examinations, during the 7 h time frame of the study (Additional file 8: Figure S8). The same was accounted for blood (data not shown). The tracer accumulation in kidneys reflects a combination of SSTR binding, excretion and reabsorption. Although SSTR_2_ receptors are expressed in vasa recta [32], glomeruli, tubular cells of the cortex and the ascending limb of Henle’s [33], the tracer excretion and reabsorption most probably mask the specific binding on the various cells in the kidney. This is also described in the report by Rolleman et al. in which two out of eight patients showed similar renal activity on ^111^In-DTPA^0^-octreotide scintigraphy, whether they were with or without SSA medication [34]. Forrer et al. recognize that although cells from the bone marrow express SSTR, the receptor density is so low that the uptake primarily reflects the blood content and is unaffected by the cellular tracer accumulation [35]. This explanation we believe is valid for our finding of similar blood and bone marrow SUV following octreotide administration.

We previously demonstrated the impact of the peptide mass on the tumor and normal tissue uptake in NET patients, undergoing three consecutive ^68^Ga-DOTATOC-PET/CT examinations during the same day—^68^Ga-DOTATOC only (10 µg), ^68^Ga-DOTATOC + 50 µg intravenous octreotide and ^68^Ga-DOTATOC + 500 µg intravenous octreotide—with subsequently decreasing normal organ SUV from the first to the third examination. However, in tumors following an initial increase from the first to the second examination, there was a decrease following injection of 500 µg of octreotide at the third examination [23]. In this previous study, the remaining activity from the previous examinations is not accounted for, neither is the effect of the previously administered peptide, and nor is receptor recirculation considered*.* The findings of Velikyan et al. were yet later confirmed in subsequent trials, as ongoing SSA medication in connection with ^68^Ga-DOTATATE-PET/CT does not hamper tumor imaging. Conversely, medication with long-acting SSAs increases the differences in tracer uptake between NETs and normal organ and consequently improves the tumor-to-normal tissue contrast [19–22]. As opposed to other receptor systems, generally showing downregulation, it has been shown that long-term continuous SSTR stimulation results in upregulation and an increase in the receptor amount in vivo [16]. Whether the rate of de novo synthesis and restoration of receptor expression in tumors differ from that in normal tissues needs to be studied. Another important factor is the so-called tumor sink effect [19], i.e., that in patients with a large tumor load with high SSTR expression, most of the preparation at PRRT is taken up by the tumor and little by the normal tissues. Thus, the total amount of SSTR in the tumor load also needs to be considered, and based on the previous experiment (Velikyan et al. Nucl Med Biol), a high amount of peptide is more beneficial when the total amount of tumor SSTR is high and vice versa.

Our present study findings indicate an application for PRRT that a favorable effect may be achieved by preloading with intravenous SSA about 5–7 h before start of PRRT, in the time frame between depleted normal organ receptors and reappeared tumor SSTRs, thus increasing the amount of available radioactive ligand for binding to the tumor SSTRs. Continuation of long-acting SSA medication, rather than pausing the treatment before PRRT cycles, may add to this effect as might an increased amount of peptide in the therapeutic preparation. The optimal strategy, in this regard, needs to be established and warrants further studies. Unfortunately, from a strictly tissue uptake point of view, administering additional peptide will not improve the situation for the kidneys, nor for the bone marrow (reflecting the radioactivity concentration in blood) [35, 36]. However, in patients with a large tumor load and high SSTR, expression, high amounts of peptide at PRRT may possibly enhance the tumor sink effect, decreasing the radiation dose to the kidneys and bone marrow.

An obvious limitation of the present work is the small number of included patients. This is a logistically challenging protocol that is very demanding for the patients, who will spend a lot of time in the clinic when undergoing their first PRRT session. Hence, we felt the consistent results across subjects permitted us to limit the study to this small group. The genuine clinical conditions were a strength of our study, including patients progressing on treatment with long-acting SSAs and scheduled for PRRT, a treatment for which the effects of the amount of administered peptide, as assessed in our study, is very pertinent. Another strength was that the study conditions were kept as constant as possible between patients, with virtually the same dosing intervals for the long-acting SSA medication before the baseline examination and before the ^68^Ga-DOTATOC-PET/CT on the day of the experiment, respectively, and that the study examinations utilized the same PET/CT scanner. Although three of the patients received the same SSA, their dosing intervals varied between one and four weeks. Also, because of logistical reasons, the interval between the screening examination and ^68^Ga-DOTATOC-PET/CT on the study day varied from 1 to 3, 5 months. As discussed previously, in this experiment, the potential impact of tumor load could not be evaluated, both because of the small number of subjects and since tumor load was moderate and fairly similar in three of the patients, and low in the fourth subject.

## Conclusions

The SSTR recovery in NETs appears within a shorter time frame compared to that in normal parenchymal abdominal organs (liver, spleen and pancreas). This opens the possibility to protect normal tissues during PRRT by administering a single dose of cold peptide hours before PRRT and most likely improves the availability of the therapeutic preparation to the tumors.

## Supplementary Information

**Additional file 1. Figure S1:** Tumor SUV at baseline (WB 0), WB 1 (0h), WB 2 (4h) and WB 3 (7h) for patient 1 (A), patient 2 (B), patient 3 (C) and patient 4 (D). Each color represents one tumor

**Additional file 2. Figure S2:** Scatter dot plots of (A) tumor SUV at baseline (WB 0), WB 1 (0h), WB 2 (4h) and WB 3 (7h) and (B) SUV normalized SUV where the tumor SUV at WB 1, 2 and 3 is normalized against tumor SUV at WB 0. The two largest tumors per patient were selected. The solid lines represent median and interquartile range and the dotted line (B) SUV ratio of 1. Significant decreases (P < 0.05) were found in tumor SUV between WB 0 and WB 1, however, not between WB 0 and WB2, and WB 0 and WB 3 (P > 0.05). Significant increase was found in normalized SUV between WB 1(0h) and WB 2 (4h) and WB 1(0h) and WB 3 (7h) but not between WB 2 and WB 3. Thus, the normalized tumor SUV returned to baseline levels at 4h and 7h

**Additional file 3. Figure S3:** Scatter dot plots of tumor SUV at WB 0, 1, 2 and 3 in (A) small (>1 to < 4 mL) and (B) large (≥ 4 mL) tumors. The solid line represents median and interquartile range. No significant increase (P > 0.05) was found in tumor SUV in small or large tumors between WB 0 and WB 1–3 except in between WB 0 and WB 1 in large tumors (P < 0.05). No significant increase (P > 0.05) was found in tumor SUV in small tumors between WB 1 and WB 2 and 3, respectively. For large tumors, significant decrease was found between WB 1 and WB 2 and 3, respectively (P < 0.05)

**Additional file 4. Figure S4:** Scatter dot plots of tumor SUV at WB 1, 2 and 3 SUV normalized against WB 0 in (A) small (>1 to < 4 mL) and (B) large (≥ 4 mL) tumors. The solid line represents median and interquartile range and the dotted line SUV ratio of 1. Significant increase was found (P < 0.05) in large tumors between WB 1 and WB 2 and WB 1 and WB 3 when normalizing against WB 0 (B), but not in small tumors (A)

**Additional file 5. Figure S5:** Scatter dot plots of tumor Ki at the first, second and third dynamic scan. The solid line represents median and interquartile range. The two largest tumors per patient were selected. Significant increase (P < 0.05) was found in tumor Ki between the first and second and first and thirds dynamic examination, however, not between the second and third scan (P > 0.05)

**Additional file 6. Figure S6:** Scatter dot plots of tumor Ki at the first, second and third dynamic scan. The solid line represents median and interquartile range. The tumors are divided into (A) small (>1 to < 4 mL) and (B) large (≥ 4 mL) tumors. For the large tumors, significant increase (P < 0.05) was found in tumor Ki between the first and second and first and thirds dynamic scan (P > 0.05), but not in small tumors

**Additional file 7. Figure S7:** Normal organs presented per patient regarding SUV at baseline (WB 0), WB 1 (0h), WB 2 (4h) and WB 3 (7h) in (A), liver (B) pancreas and (C) spleen. Normalized SUV, where SUV in WB 1 (0h), WB 2 (4h) and WB 3 (7h) is normalized against SUV at WB 0 (SUV ratio) in (D) liver, (E) pancreas and (F) spleen. The solid lines represent median and range and the dotted line the normalized SUV value of 1. Following the initial SUV drop from baseline to WB1, a slow recovery was found during WB 2 and WB 3, but not back to baseline levels

**Additional file 8. Figure S8:** Scatter dot plots of SUV at WB 0, 1, 2 and 3 in (A) right kidney (Dx), (B) Left kidney (Sin) and (C) bone marrow. Normalized SUV—where SUV in WB 1, 2 and 3 is normalized against SUV at WB is shown for (D) right kidney, (E) Left kidney and (F) bone marrow. The solid line represents median and interquartile range and the dotted line (B) SUV ratio of 1. The SUV and the normalized SUV were similar between scans for both kidneys and bone marrow (P > 0.05)

**Additional file 9. Figure S9:** Correlation between (A) tumor Ki and tumor SUV and between (B) tumor Ki and tumor-to-blood ratio (TBR) during all three dynamic scans. The two largest tumors per patient were selected. Solid line represents linear regression fits, and dashed line are 95% confidence band of these fits. R2 represents the square of Pearson correlation

**Additional file 10. Figure S10:** Correlation between Ki and SUV in (A) small (>1 to <4mL) and (B) large (≥ 4mL) tumors during all three dynamic scans. Solid line represents linear regression fits and dashed line are 95% confidence band of these fits. R2 represents the square of Pearson correlation

**Additional file 11. Figure S11:** Correlation between Ki and tumor-to-blood ratio (TBR) in(A) small (>1 to < 4mL) and (B) large (≥ 4mL) tumors during all three dynamic scans. Solid line represents linear regression fits, and dashed lines are 95% confidence band of these fits. R2 represents the square of Pearson correlation.

**Additional file 12. Figure S12:** Tumor Ki at WB 1 (0h), WB 2 (4h) and WB 3 (7h) for patient 1 (A), patient 2 (B), patient 3 (C) and patient 4 (D). Each color represents one tumor

## Data Availability

The dataset used and/or analyzed during the current study are available from the corresponding author on reasonable request.
