# Supplementary figures and images for: Receptor depletion and recovery in small-intestinal neuroendocrine tumors and normal tissues after administration of a single intravenous dose of octreotide measured by 68Ga-DOTATOC PET/CT
Source: EJNMMI Res. 2021 Nov 25;11:118. doi: 10.1186/s13550-021-00860-0 (PMC8617112; doi:10.1186/s13550-021-00860-0)

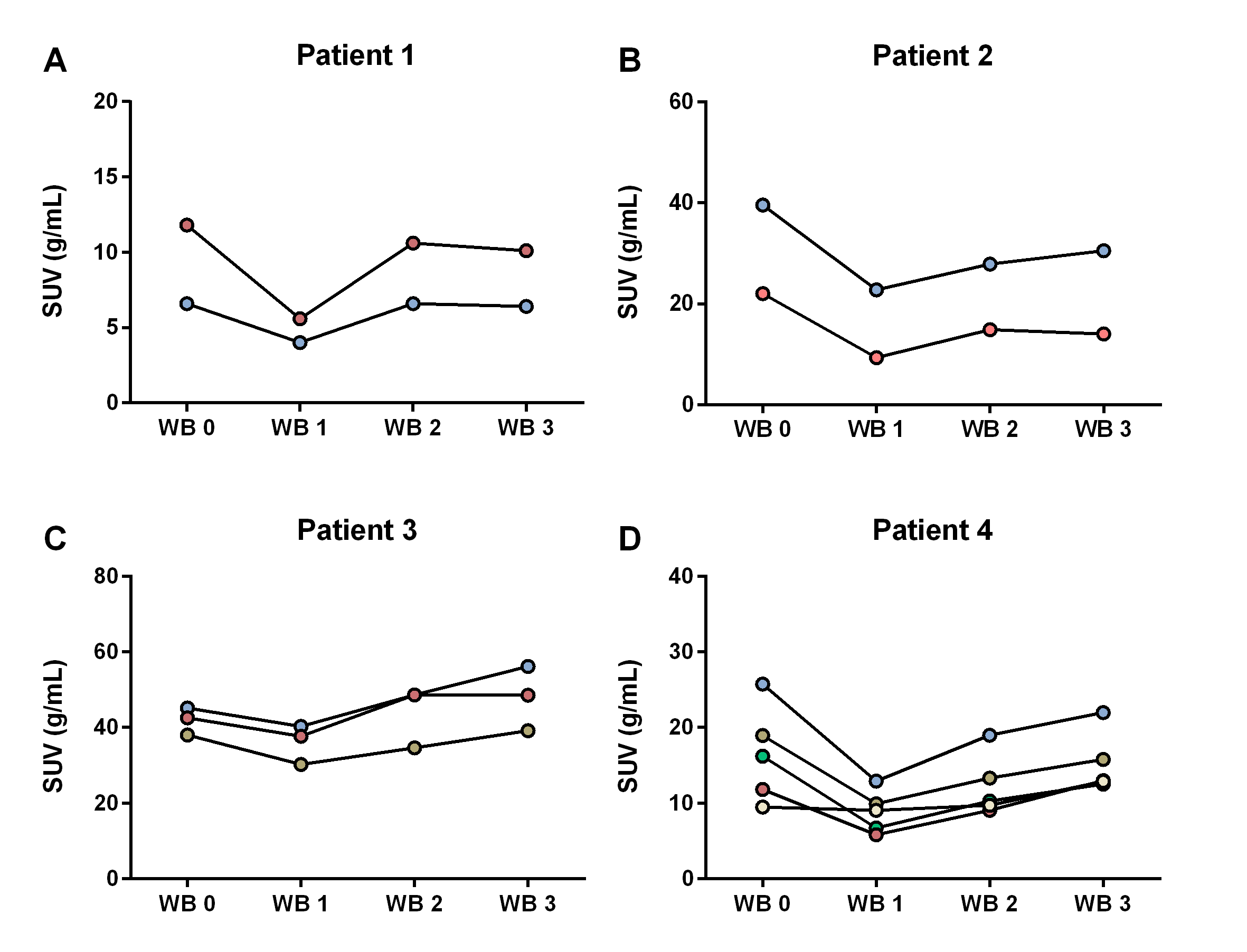

Supplement: Supplementary file 1 — Additional file 1. Figure S1: Tumor SUV at baseline (WB 0), WB 1 (0h), WB 2 (4h) and WB 3 (7h) for patient 1 (A), patient 2 (B), patient 3 (C) and patient 4 (D). Each color represents one tumor [file 13550_2021_860_MOESM1_ESM.tif]

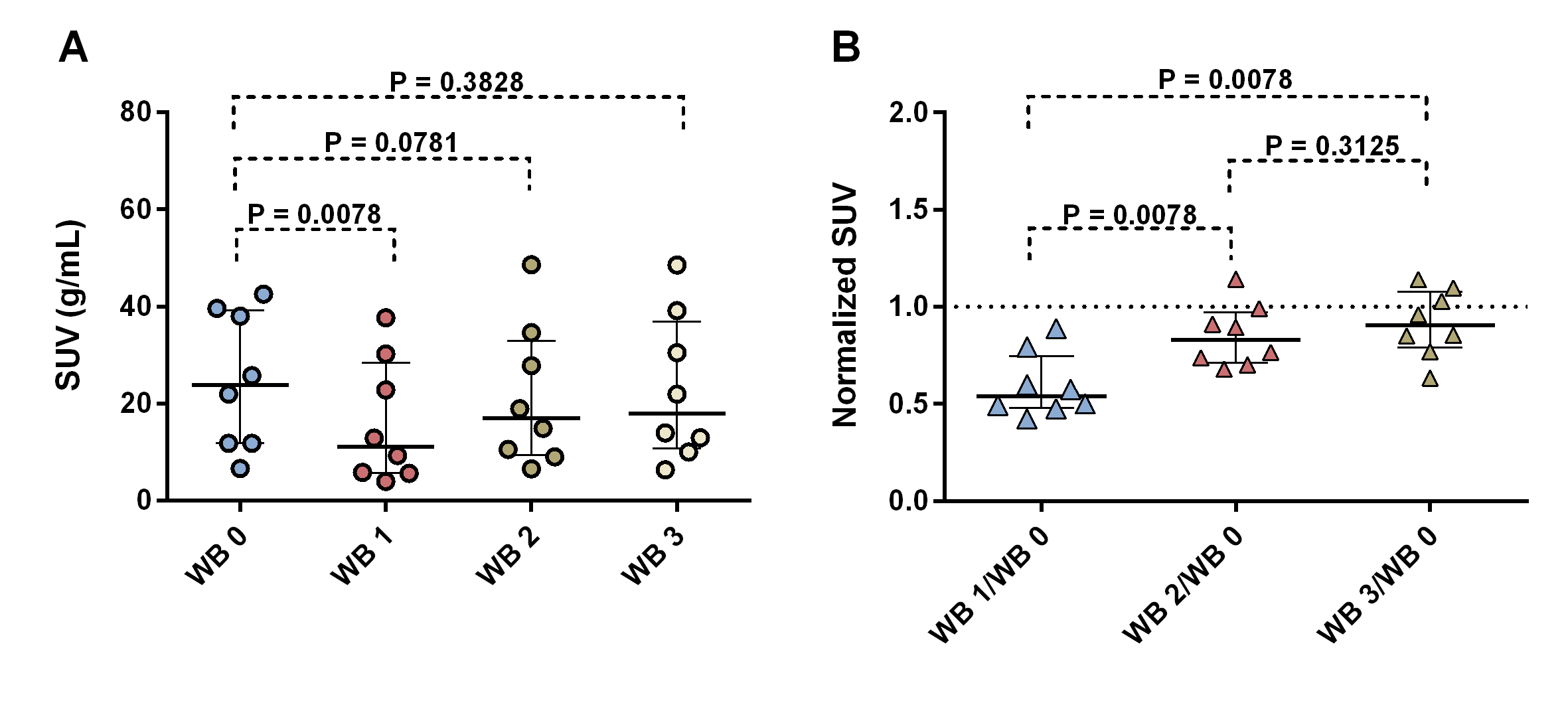

Supplement: Supplementary file 2 — Additional file 2. Figure S2: Scatter dot plots of (A) tumor SUV at baseline (WB 0), WB 1 (0h), WB 2 (4h) and WB 3 (7h) and (B) SUV normalized SUV where the tumor SUV at WB 1, 2 and 3 is normalized against tumor SUV at WB 0. The two largest tumors per patient were selected. The solid lines represent median and interquartile range and the dotted line (B) SUV ratio of 1. Significant decreases (P < 0.05) were found in tumor SUV between WB 0 and WB 1, however, not between WB 0 and WB2, and WB 0 and WB 3 (P > 0.05). Significant increase was found in normalized SUV between WB 1(0h) and WB 2 (4h) and WB 1(0h) and WB 3 (7h) but not between WB 2 and WB 3. Thus, the normalized tumor SUV returned to baseline levels at 4h and 7h [file 13550_2021_860_MOESM2_ESM.tif]

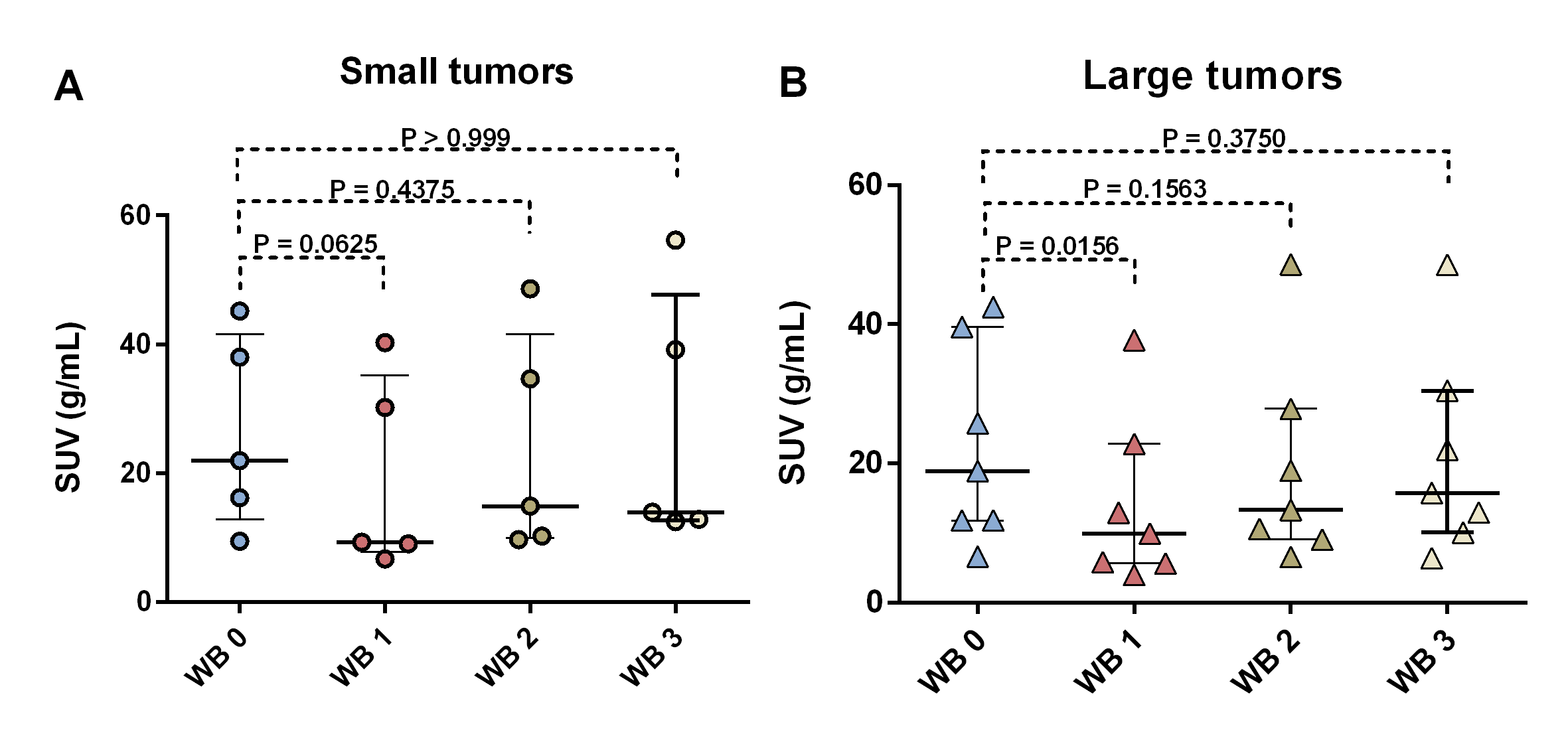

Supplement: Supplementary file 3 — Additional file 3. Figure S3: Scatter dot plots of tumor SUV at WB 0, 1, 2 and 3 in (A) small (>1 to < 4 mL) and (B) large (≥ 4 mL) tumors. The solid line represents median and interquartile range. No significant increase (P > 0.05) was found in tumor SUV in small or large tumors between WB 0 and WB 1–3 except in between WB 0 and WB 1 in large tumors (P < 0.05). No significant increase (P > 0.05) was found in tumor SUV in small tumors between WB 1 and WB 2 and 3, respectively. For large tumors, significant decrease was found between WB 1 and WB 2 and 3, respectively (P < 0.05) [file 13550_2021_860_MOESM3_ESM.tif]

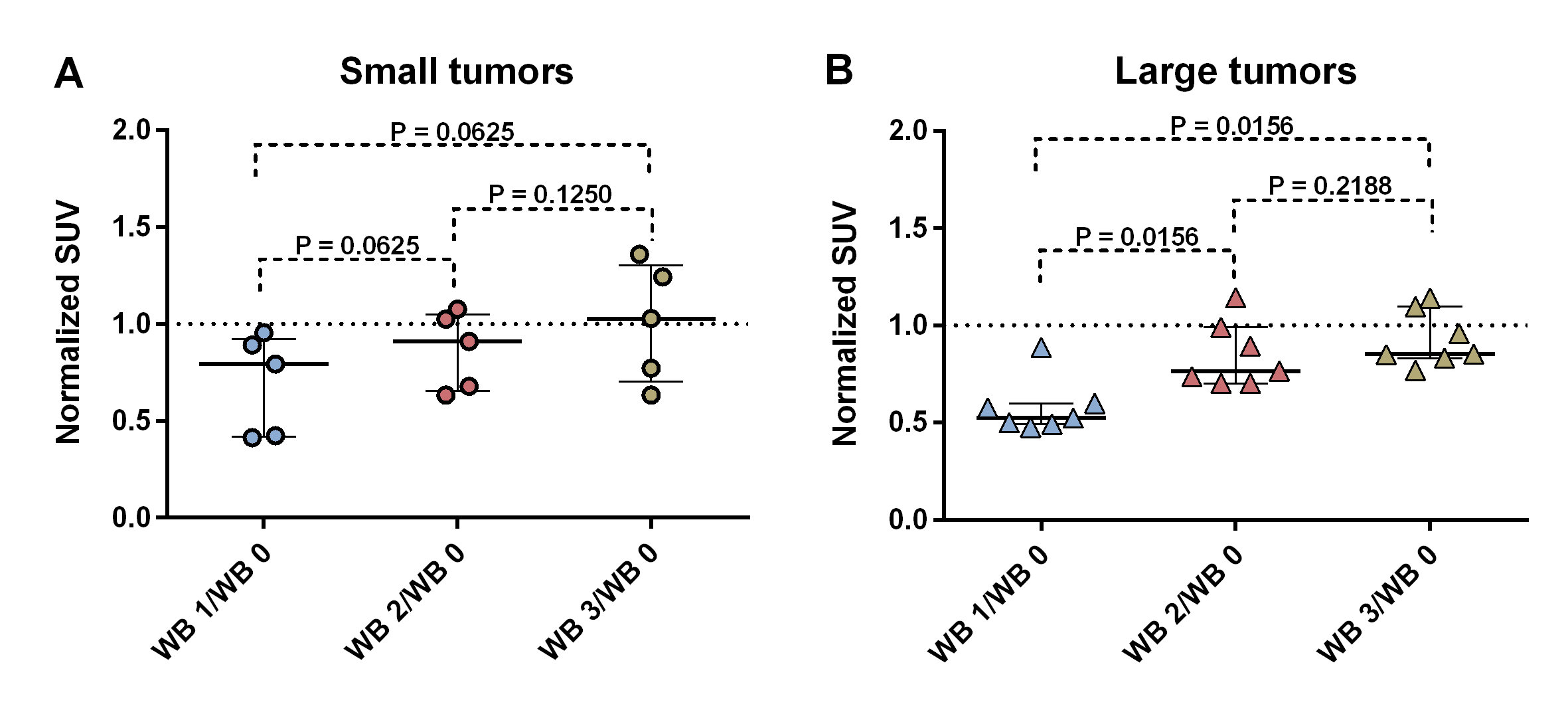

Supplement: Supplementary file 4 — Additional file 4. Figure S4: Scatter dot plots of tumor SUV at WB 1, 2 and 3 SUV normalized against WB 0 in (A) small (>1 to < 4 mL) and (B) large (≥ 4 mL) tumors. The solid line represents median and interquartile range and the dotted line SUV ratio of 1. Significant increase was found (P < 0.05) in large tumors between WB 1 and WB 2 and WB 1 and WB 3 when normalizing against WB 0 (B), but not in small tumors (A) [file 13550_2021_860_MOESM4_ESM.tif]

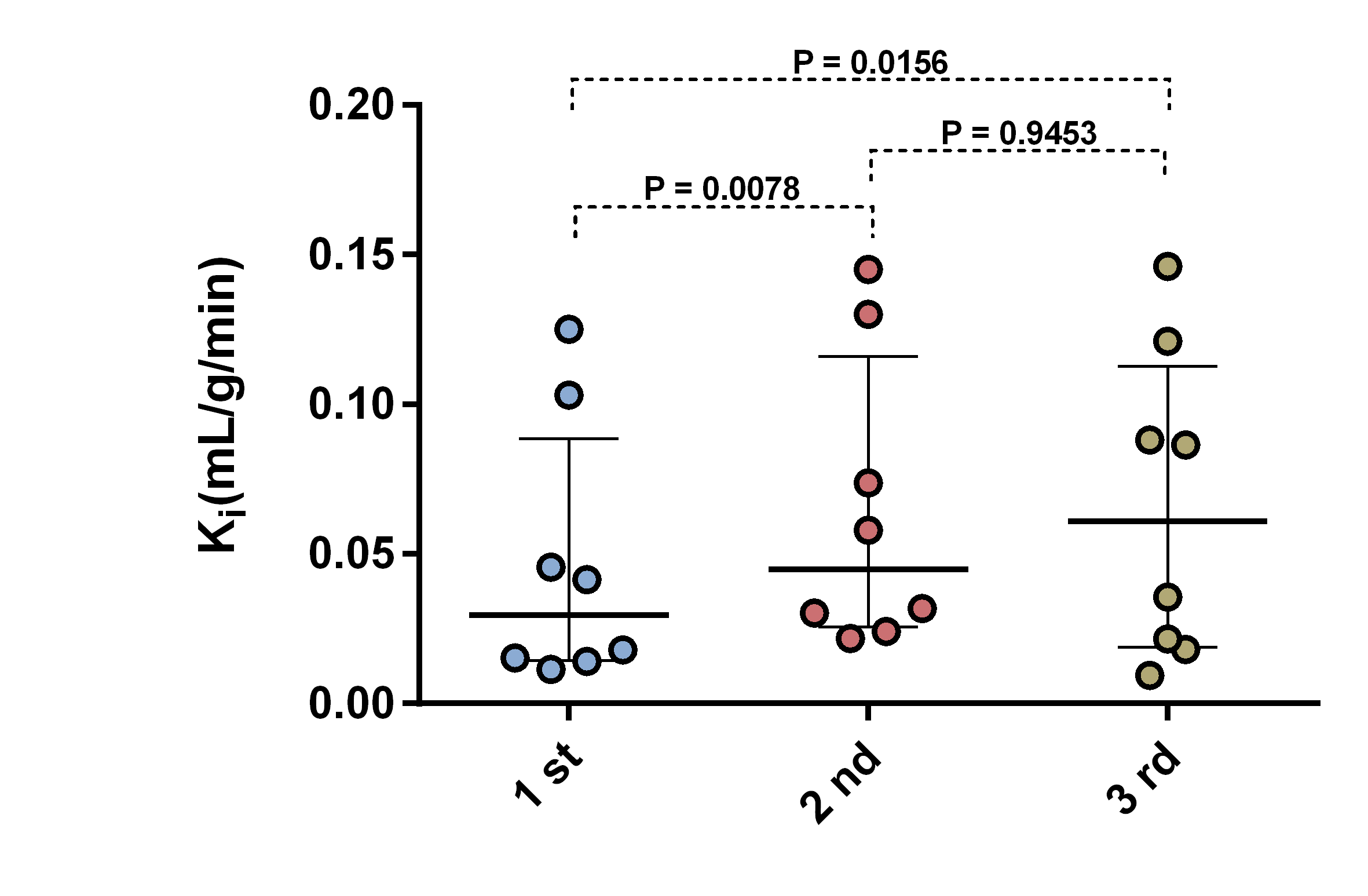

Supplement: Supplementary file 5 — Additional file 5. Figure S5: Scatter dot plots of tumor Ki at the first, second and third dynamic scan. The solid line represents median and interquartile range. The two largest tumors per patient were selected. Significant increase (P < 0.05) was found in tumor Ki between the first and second and first and thirds dynamic examination, however, not between the second and third scan (P > 0.05) [file 13550_2021_860_MOESM5_ESM.tif]

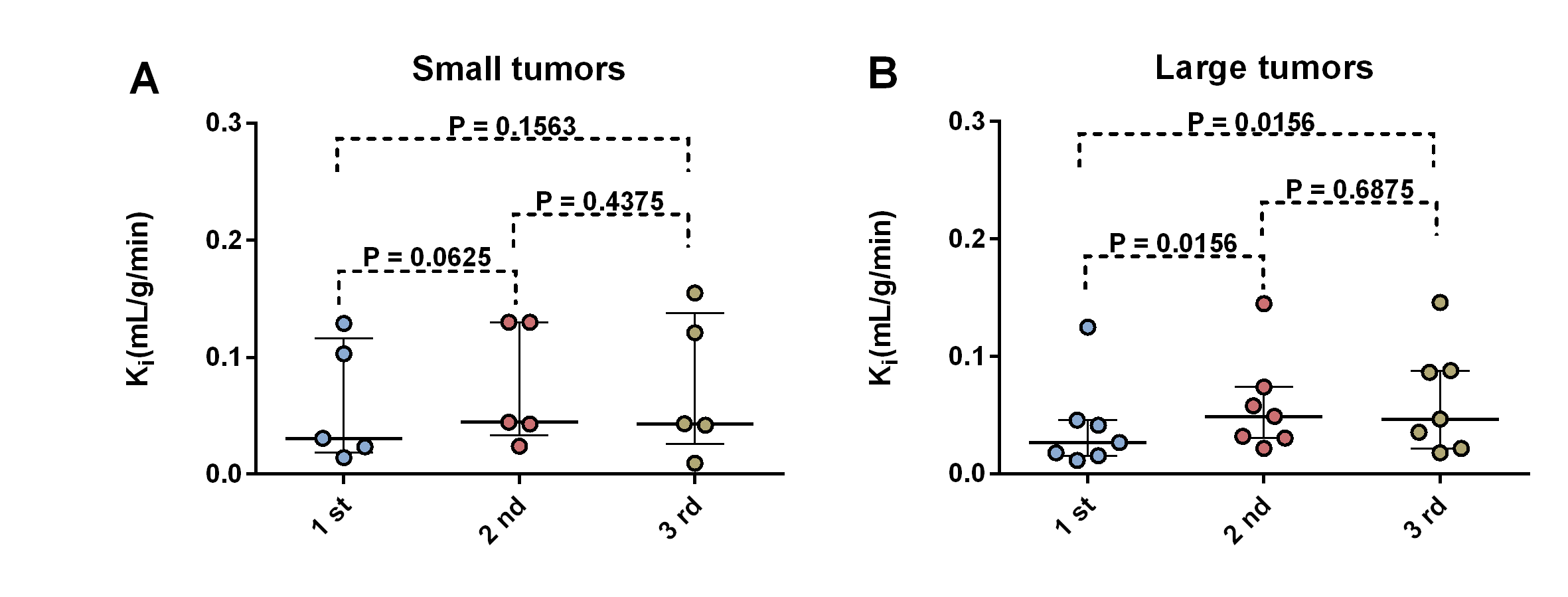

Supplement: Supplementary file 6 — Additional file 6. Figure S6: Scatter dot plots of tumor Ki at the first, second and third dynamic scan. The solid line represents median and interquartile range. The tumors are divided into (A) small (>1 to < 4 mL) and (B) large (≥ 4 mL) tumors. For the large tumors, significant increase (P < 0.05) was found in tumor Ki between the first and second and first and thirds dynamic scan (P > 0.05), but not in small tumors [file 13550_2021_860_MOESM6_ESM.tif]

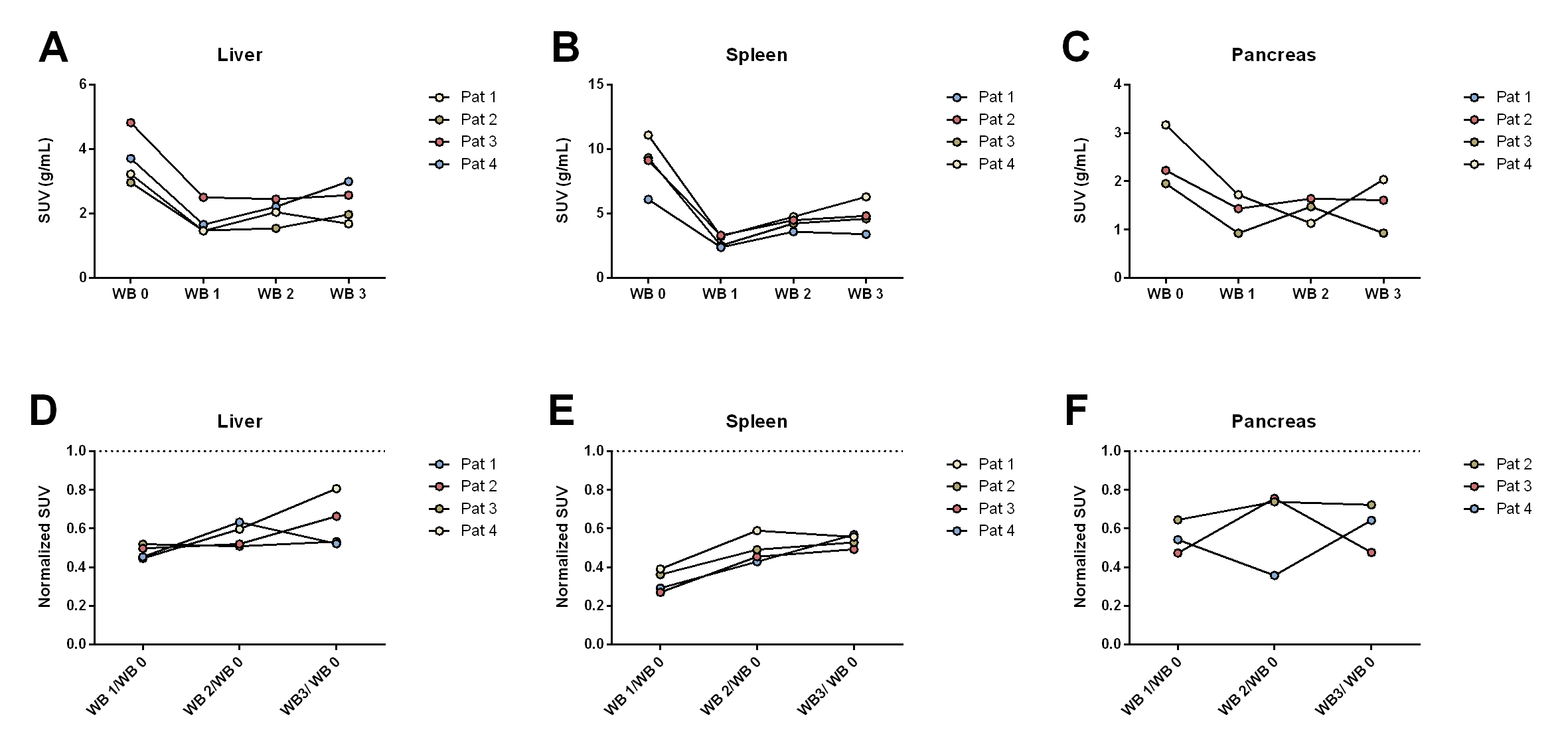

Supplement: Supplementary file 7 — Additional file 7. Figure S7: Normal organs presented per patient regarding SUV at baseline (WB 0), WB 1 (0h), WB 2 (4h) and WB 3 (7h) in (A), liver (B) pancreas and (C) spleen. Normalized SUV, where SUV in WB 1 (0h), WB 2 (4h) and WB 3 (7h) is normalized against SUV at WB 0 (SUV ratio) in (D) liver, (E) pancreas and (F) spleen. The solid lines represent median and range and the dotted line the normalized SUV value of 1. Following the initial SUV drop from baseline to WB1, a slow recovery was found during WB 2 and WB 3, but not back to baseline levels [file 13550_2021_860_MOESM7_ESM.tif]

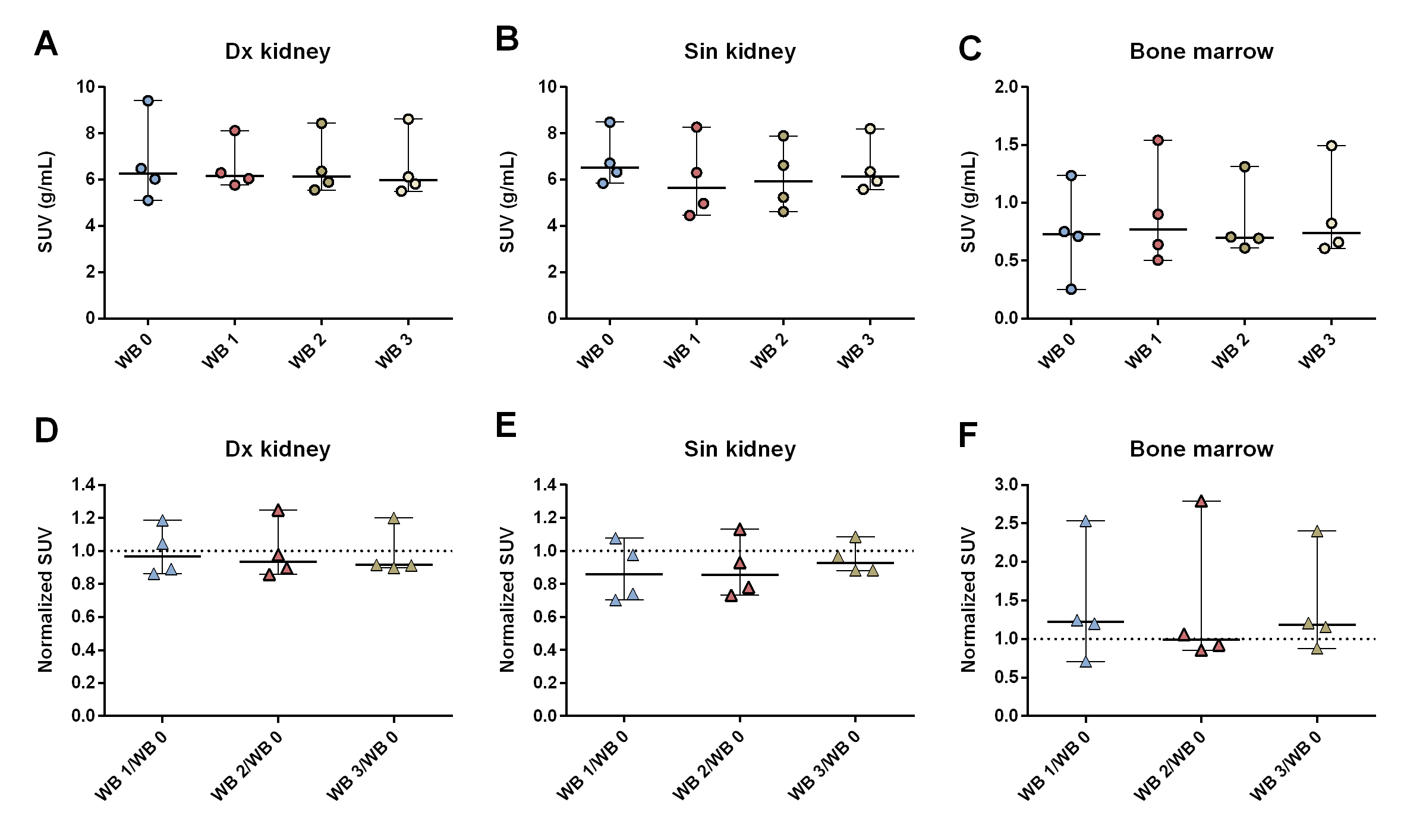

Supplement: Supplementary file 8 — Additional file 8. Figure S8: Scatter dot plots of SUV at WB 0, 1, 2 and 3 in (A) right kidney (Dx), (B) Left kidney (Sin) and (C) bone marrow. Normalized SUV—where SUV in WB 1, 2 and 3 is normalized against SUV at WB is shown for (D) right kidney, (E) Left kidney and (F) bone marrow. The solid line represents median and interquartile range and the dotted line (B) SUV ratio of 1. The SUV and the normalized SUV were similar between scans for both kidneys and bone marrow (P > 0.05) [file 13550_2021_860_MOESM8_ESM.tif]

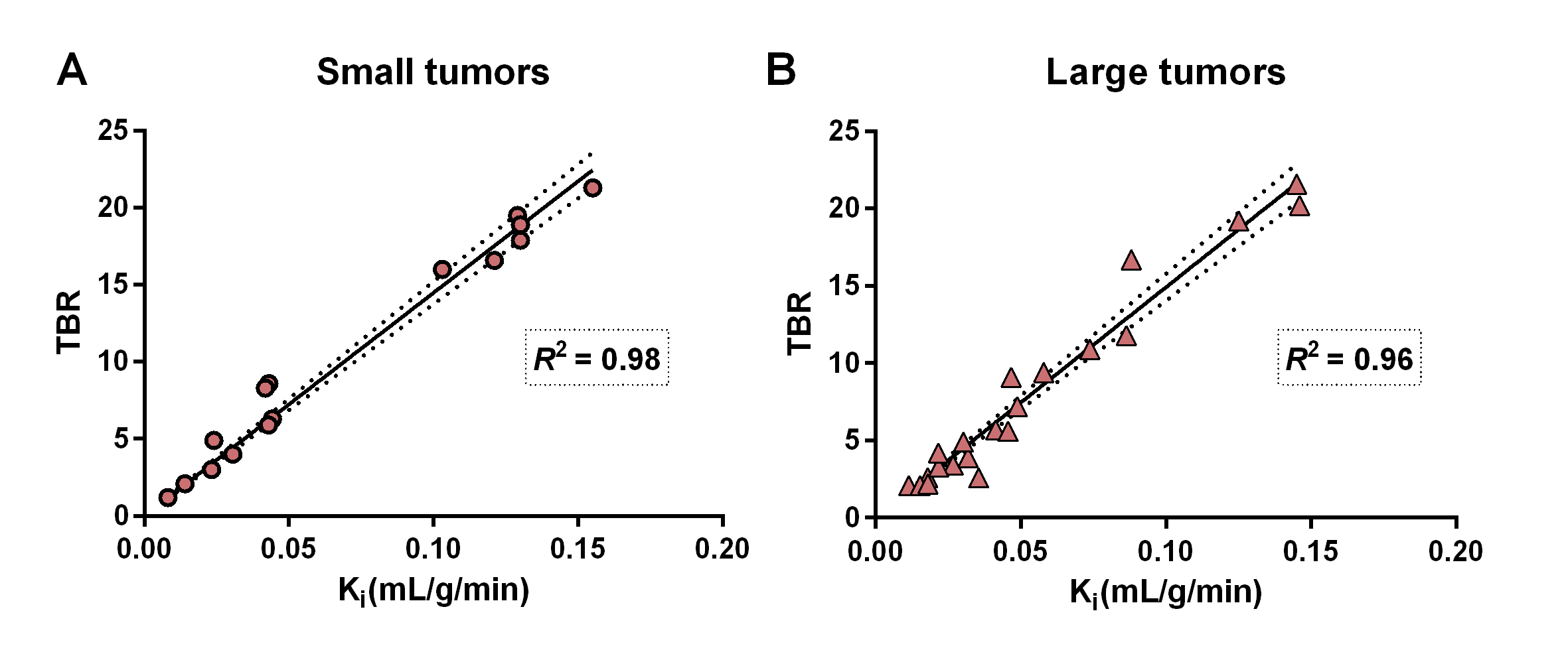

Supplement: Supplementary file 11 — Additional file 11. Figure S11: Correlation between Ki and tumor-to-blood ratio (TBR) in(A) small (>1 to < 4mL) and (B) large (≥ 4mL) tumors during all three dynamic scans. Solid line represents linear regression fits, and dashed lines are 95% confidence band of these fits. R2 represents the square of Pearson correlation. [file 13550_2021_860_MOESM11_ESM.tif]

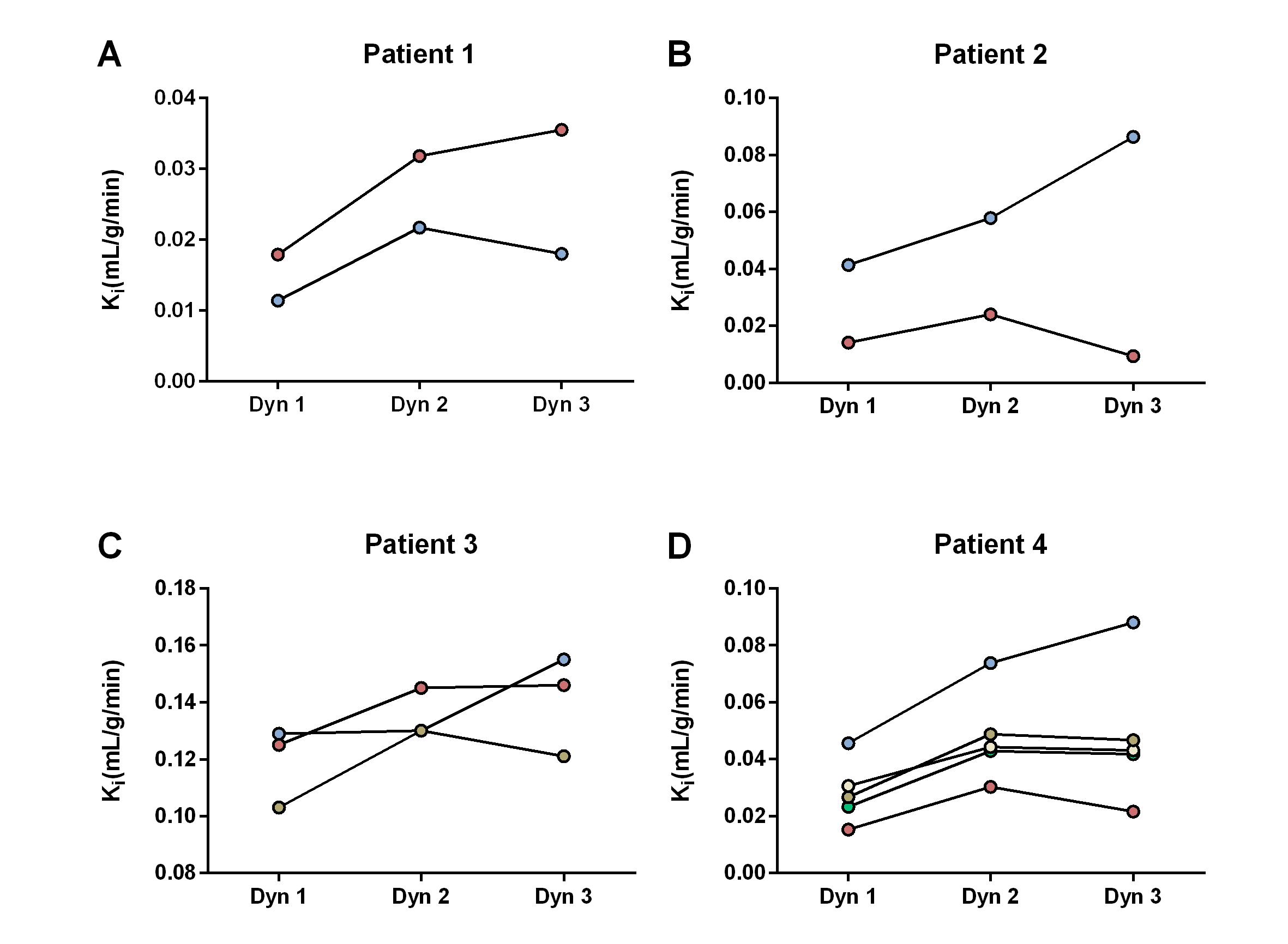

Supplement: Supplementary file 12 — Additional file 12. Figure S12: Tumor Ki at WB 1 (0h), WB 2 (4h) and WB 3 (7h) for patient 1 (A), patient 2 (B), patient 3 (C) and patient 4 (D). Each color represents one tumor [file 13550_2021_860_MOESM12_ESM.tif]
